# A Systematic Workflow for Compliance Testing of Emerging International Classwide Restrictions on PFAS

**DOI:** 10.1021/acs.est.4c06570

**Published:** 2024-08-14

**Authors:** Robin Vestergren, Anders Appelblom, Simona A. Bălan, Sicco H. Brandsma, Thomas A. Bruton, Ian T. Cousins, Jeremy R. Gauthier, Audun Heggelund, Jenny Ivarsson, Anna Kärrman, Lisa Melymuk, Chijioke Olisah, Amanda Rosen, Eleni K. Savvidou, Steffen Schellenberger, Lisa Skedung, Petteri Talasniemi, Tonie Wickman, Jonathan Zweigle, Christian Zwiener, Jonathan P. Benskin

**Affiliations:** † Swedish Chemicals Agency (KEMI), 17266 Stockholm, Sweden; ‡ California Department of Toxic Substances Control, Berkeley, California 94710, United States; § Amsterdam Institute for Life and Environment (A-LIFE), Vrije Universiteit Amsterdam, 1081 HV Amsterdam, The Netherlands; ∥ California Department of Toxic Substances Control, Sacramento, California 95814, United States; ⊥ Department of Environmental Science, Stockholm University, 10691 Stockholm, Sweden; # Department of Chemistry, University of Toronto, Toronto, ON M5S 3H6, Canada; 7 Norwegian Environment Agency, Box 5672, Torgarden, N-7485 Trondheim, Norway; 8 School of Science and Technology, Örebro University, 70182 Örebro, Sweden; 9 RECETOX, Faculty of Science, Masaryk University, 61137 Brno, Czech Republic; 10 RISE Research Institutes of Sweden AB, Environment and Sustainable Chemistry Unit, 11428 Stockholm, Sweden; 11 RISE Research Institutes of Sweden AB, Department Materials and Surface Design, 11428 Stockholm, Sweden; 12 Finnish Safety and Chemicals Agency (Tukes), Box 66, 00521 Helsinki, Finland; 13 RISE Research Institutes of Sweden AB, The Swedish Centre for Chemical Substitution, 11428 Stockholm, Sweden; 14 Environmental Analytical Chemistry, Department of Geosciences, University of Tübingen, 72076 Tübingen, Germany

**Keywords:** PFAS, compliance testing, analytical methods, classwide restrictions

The poorly reversible risks to human health and ecosystems from contamination with per- and polyfluoroalkyl substances (PFAS) have led many researchers and regulators worldwide to call for a classwide ban of these so-called forever chemicals. As part of the EU Chemicals Strategy for Sustainability, the national authorities of five European countries submitted a broad restriction proposal on PFAS under REACH in January 2023. This restriction proposal is unique in its scope by including the vast majority of uses for >10 000 substances that meet the OECD definition of PFAS.^1^ In parallel, several countries and multiple states in the United States have proposed or enacted broad PFAS restrictions for all non-essential uses or for specific uses and reporting requirements for a range of consumer products. Although the regulatory frameworks underpinning these restrictions contain many differences, the proposed restrictions have the common objective to ban the intentional use of all PFAS and thus avoid regrettable substitution with other PFAS. Given that the proposed restrictions apply to chemical products and articles (both hereafter termed simply “products”) that are imported from other states, countries, or regions, they may also trigger substitution and an increased demand for supply chain information on a global level. Direct communication with manufacturers and distributors is typically the primary approach for companies to ensure compliance with chemical regulations. Nevertheless, companies and authorities require reliable analytical methods to independently verify supply chain information and capture products that are noncompliant with PFAS restrictions.

A major challenge for compliance testing stems from the sheer number and structural diversity of PFAS, making it impossible for a single analytical method to quantify all PFAS individually. There are, however, a growing number of analytical methods that can indicate the presence of PFAS by leveraging certain characteristics of these chemicals. Building on the recent advances in the analytical chemistry of PFAS, we discuss the currently available analytical methods that can inform compliance testing of PFAS in different products under different regulatory frameworks while highlighting the advantages and remaining challenges associated with these methods. We then illustrate how these methods could be applied in a three-step workflow for the implementation of the PFAS restriction proposal under REACH (Figure 1). Notably, this Viewpoint is not intended to review or comment on individual or classwide PFAS risk assessments that have been carried out by different authorities but rather to present an approach for ensuring compliance with these new laws based on recent advances in analytical chemistry.

**Figure 1:**
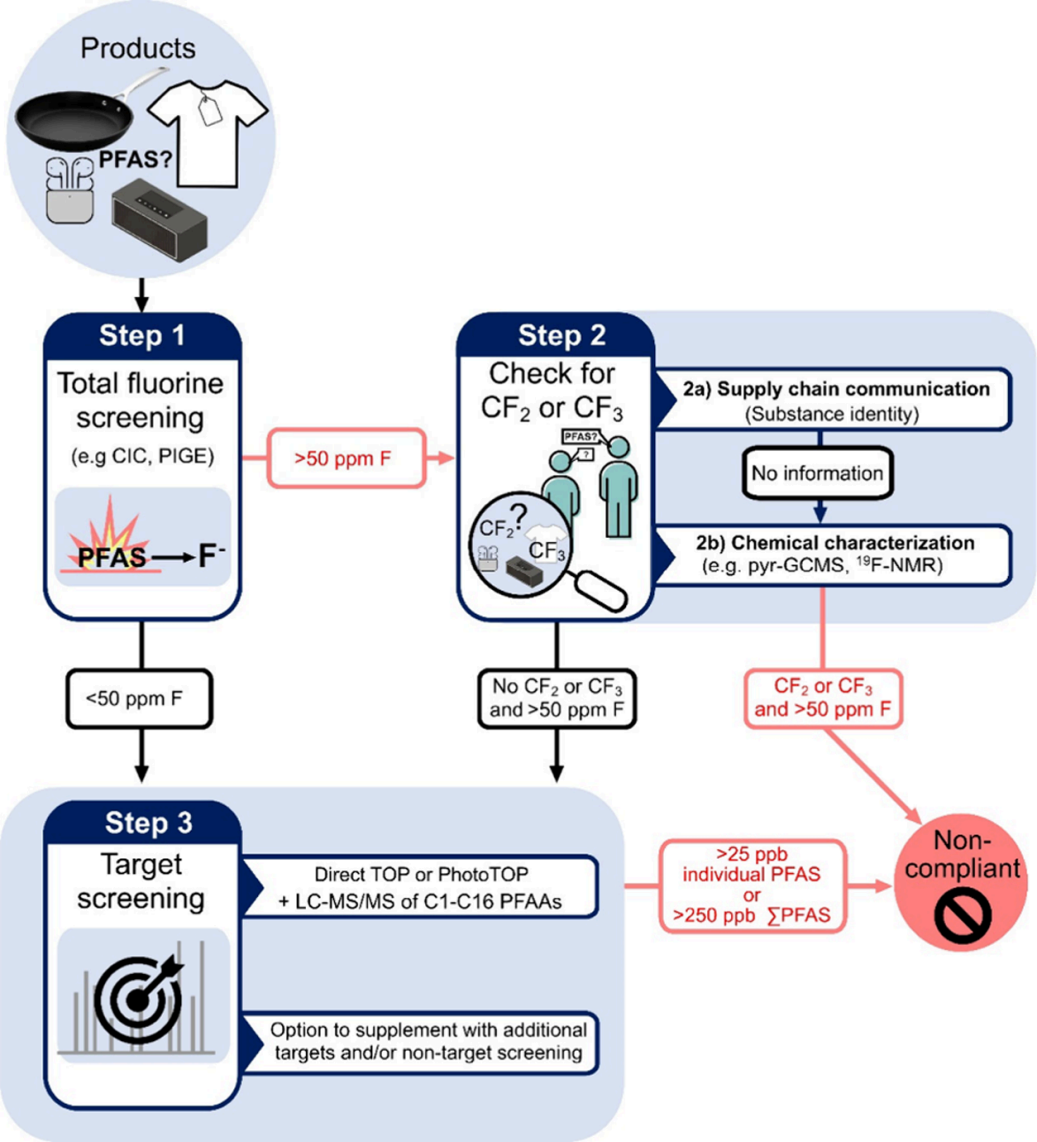
Three-step workflow that companies or authorities could implement to assess noncompliance with the proposed broad restriction of per- and polyfluoroalkyl substances (PFAS) under REACH. The submitted REACH restriction proposal has set guideline levels for both total fluorine (TF) and individual and/or sum of PFAS concentrations (ΣPFAS) with three guideline values in products: (1) 50 ppm (mg/kg) fluorine for PFAS, including polymeric PFAS; (2) 25 ppb for any PFAS measured by target analysis, excluding polymeric PFAS; and (3) 250 ppb for ΣPFAS measured by target analysis, which can optionally involve measurement of PFAS after the transformation of precursors [e.g., via the total oxidizable precursor (TOP) assay]. Because a full characterization of all commercially available PFAS, their impurities, and their degradation products is not practically feasible, this workflow is designed to efficiently identify noncompliant products using a tiered approach. Abbreviations: CIC, combustion ion chromatography; PIGE, particle-induced γ-ray emission; pyr-GCMS, pyrolysis-gas chromatography-mass spectrometry; ^19^F NMR, ^19^F nuclear magnetic resonance; LC-MS/MS, liquid chromatography-tandem mass spectrometry; PFAAs, perfluoroalkyl acids.

## Total Fluorine Screening

Screening for total fluorine (TF) provides a relatively fast and inexpensive way to assess whether PFAS may be present in a sample. A key feature of TF screening (and in contrast to extractable or adsorbable organic fluorine) is that samples are not extracted prior to analysis, making sample preparation relatively easy and allowing all PFAS to be indirectly quantified. Examples of such techniques include combustion ion chromatography (CIC) and particle-induced γ-ray emission (PIGE) spectroscopy.^2^ Additional TF methods [e.g., instrumental neutron activation analysis (INAA), X-ray photoelectron spectroscopy (XPS), and ^19^F nuclear magnetic resonance (NMR)] may also be suitable for compliance testing if they demonstrate performance across a range of matrices. The most important consideration for these methods is whether their detection limits comply with the limit values defined in the relevant regulation. Fluorine detection limits of <30% of the restriction limit value (where applicable) and a measurement uncertainty of 50% on a weight basis of the material may be reasonable criteria for successfully applying these methods as part of compliance testing. Because some methods measure bulk material concentrations (e.g., CIC) while others measure surface concentrations (e.g., PIGE), the choice of a specific technique for screening may depend on several factors, including product homogeneity and whether the limit value is defined on a mass or area basis.

## Confirmation of CF_2_ or CF_3_ Moieties

Because TF methods may be subject to false positives from inorganic fluorine or non-PFAS organofluorines, further information may be needed for products that can contain other fluorine sources besides PFAS. For example, under the proposed REACH restriction, if screening finds that the level of a product exceeds 50 ppm TF, the manufacturer, importer, or downstream user is obligated to provide proof that the measured TF originates from inorganic or non-PFAS organofluorine. The most pragmatic approach for addressing this requirement is to directly consult the supplier and obtain disclosure of any PFAS. If reliable information is not available, the presence or absence of CF_2_ or CF_3_ groups may be determined analytically. A suitable method does not necessarily need to deduce the exact chemical structure but should be suitable for robustly detecting CF_2_ or CF_3_ groups across a wide range of PFAS and products at detection limits that are ideally <30% of the restriction limit value. Importantly, the method should not require pretreatment steps (e.g., extraction), which could introduce bias,^3^ and CF_2_ or CF_3_ groups should not be produced as analytical artifacts during the analysis. Examples of methods that may be suitable for this purpose include pyrolysis-gas chromatography-mass spectrometry (pyr-GCMS) and ^19^F NMR,^4,5^ but considerable work is still required to validate these approaches for application in a regulatory context. For pyr-GCMS, users should also be aware of the potential for false positives from substances containing CF_2_ or CF_3_ groups that either do not meet the formal PFAS definition or are excluded from a restriction (see examples in ref (6)). The ^19^F NMR technique is currently predominantly available for liquid samples, and further research is needed for solid state applications.

## Quantifying Individual PFAS or the Sum of PFAS

Some restrictions include limit values for individual PFAS or the sum of PFAS (ΣPFAS) that are often several orders of magnitude lower than the detection limits of TF methods, requiring more specific analytical approaches. For example, the REACH restriction proposal includes a 25 ppb limit for individual PFAS (excluding polymers) and a 250 ppb limit for ΣPFAS measured by target analysis optionally following transformation of precursors. The rationale for these comparatively low values is that low-molecular weight PFAS often occur as impurities in products containing polymeric PFAS. Additionally, there may be cases in which low-molecular weight PFAS are intentionally added at levels close to or even below TF limit values.^7^

To address limits associated with individual PFAS or ΣPFAS, we recommend (at a minimum) measurement of C2–C16 perfluoroalkyl carboxylic acids (PFCAs) and C1–C10 perfluoroalkyl sulfonic acids (PFSAs) after the transformation of their precursors. The proposed range is based on the prevalence of these perfluoroalkyl acid (PFAA) homologues and their precursors in the environment. Methods for the transformation of PFAA precursors to PFAAs include the total oxidizable precursors (TOP) and photoTOP methods, which should be directly and quantitatively applied to the product, rather than the extracts.^3^ Moreover, in cases in which individual or ΣPFAS limits are stipulated without precursor transformation, additional analyses should be performed without TOP or photoTOP.

These methods may be supplemented with additional target substances (e.g., non-PFCA-forming PFAS such as perfluoroether carboxylic acids). The choice of targets could be based on supply chain information or prior knowledge, e.g., from technical literature on product uses and ingredients. When no prior information is available, liquid chromatography (LC)- and GC-based nontarget analytical techniques, in particular those suitable for flagging PFAS,^8,9^ may also prove useful. Here, ionization efficiency approaches offer an opportunity for quantification in the absence of standards,^10^ but the efficacy and acceptance of these approaches in a regulatory context remain unclear.

## Outlook

The workflow in Figure 1 illustrates how the methods described above could be used by manufacturers and importers or by relevant authorities to assess compliance with the proposed classwide restrictions on PFAS under REACH. Because a full characterization of all commercially available PFAS, their impurities, and their degradation products is not practically feasible, the workflow does not offer verification of a compliant product. The workflow is rather designed to efficiently identify noncompliant products using a tiered approach. As methods are developed and refined for different product categories, it is conceivable that some steps may be modified or combined to save costs and improve throughput. Other regulatory frameworks besides REACH may require only part of this workflow to assess compliance, depending on their scope.

As more jurisdictions enact PFAS-related bans, the demand for the methods discussed here will increase. However, most of these methods are currently not commercially available. We recommend that analytical laboratories focus on further developing these methods to support compliance with these new and emerging regulations around the world. To be generally accepted for compliance, the methods must be validated and standardized and demonstrate high accuracy, precision, specificity, and robustness and sufficiently low detection limits. Timely action to validate and standardize these methods will help facilitate implementation of classwide PFAS restrictions globally and ultimately pave the way for more efficient group management of chemicals in the future.
